# Supplementary figures and images for: Human Milk Oligosaccharide Utilization in Intestinal Bifidobacteria Is Governed by Global Transcriptional Regulator NagR
Source: mSystems. 2022 Sep 12;7(5):e00343-22. doi: 10.1128/msystems.00343-22 (PMC9599254; doi:10.1128/msystems.00343-22)

**A**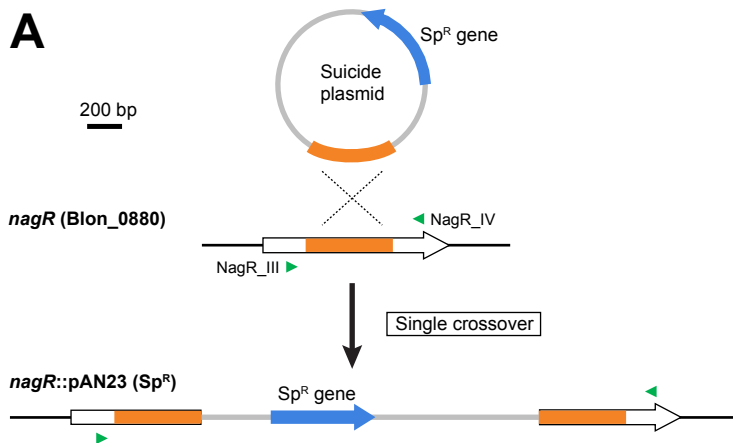**B**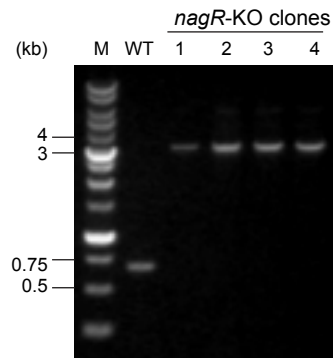**C**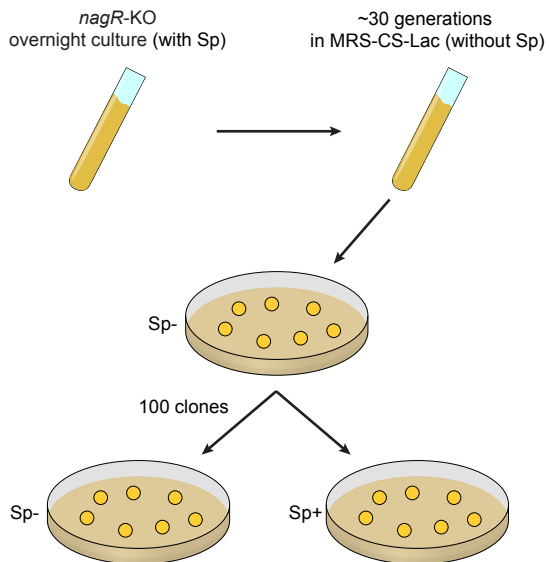

GAM plates

without Sp

with Sp

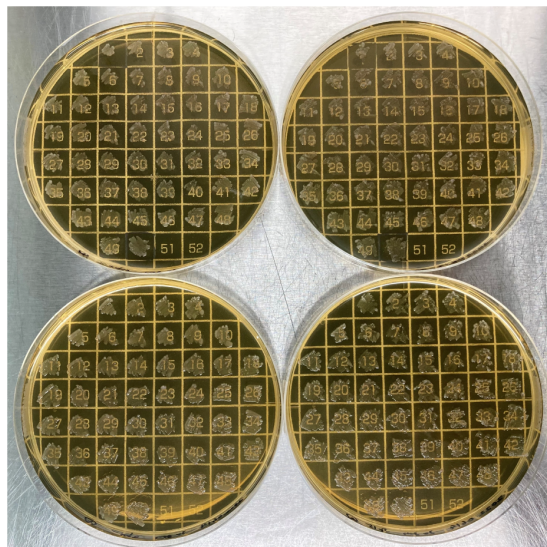

Supplement: FIG S2 [file msystems.00343-22-s0004.pdf]

**A**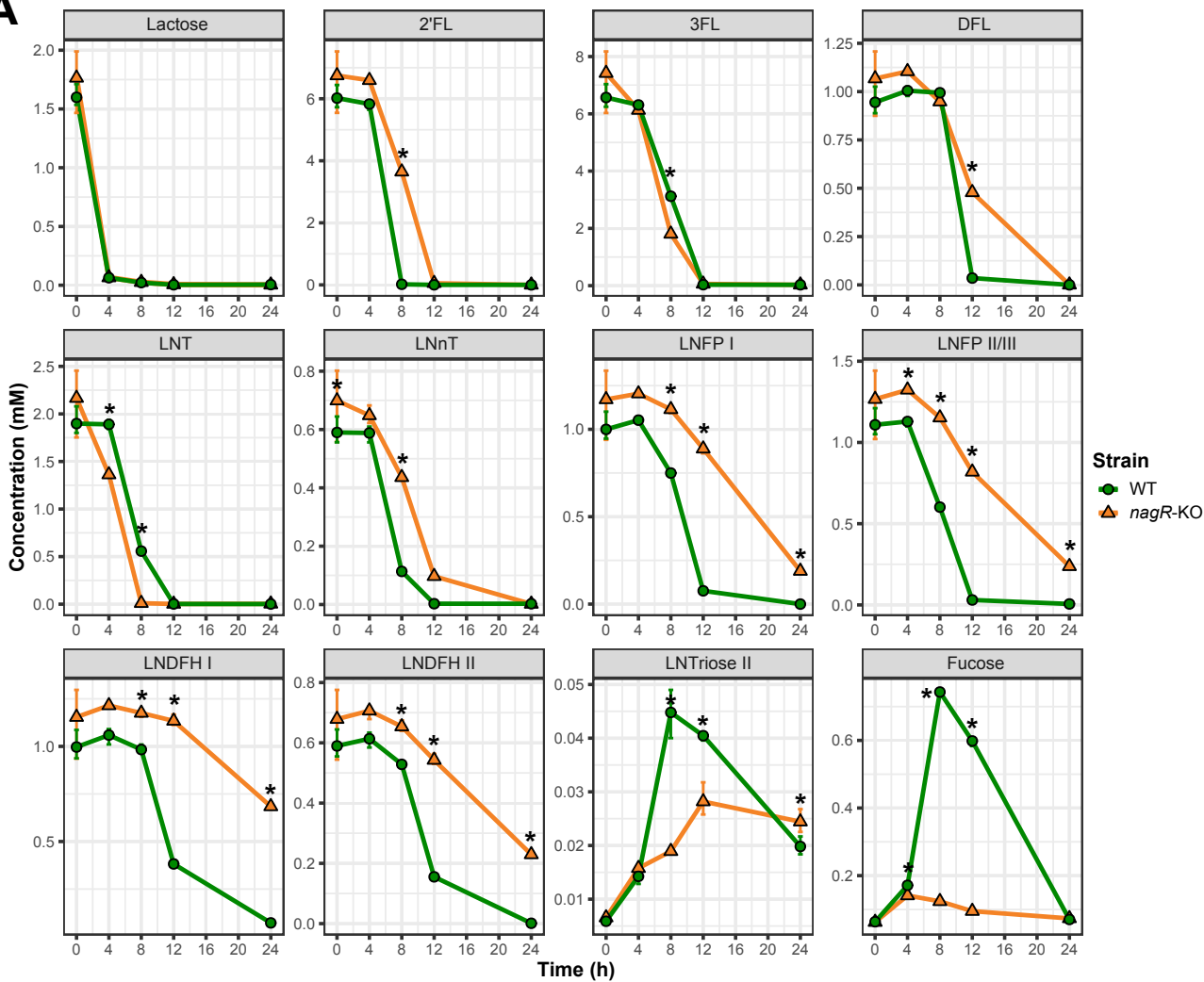**B**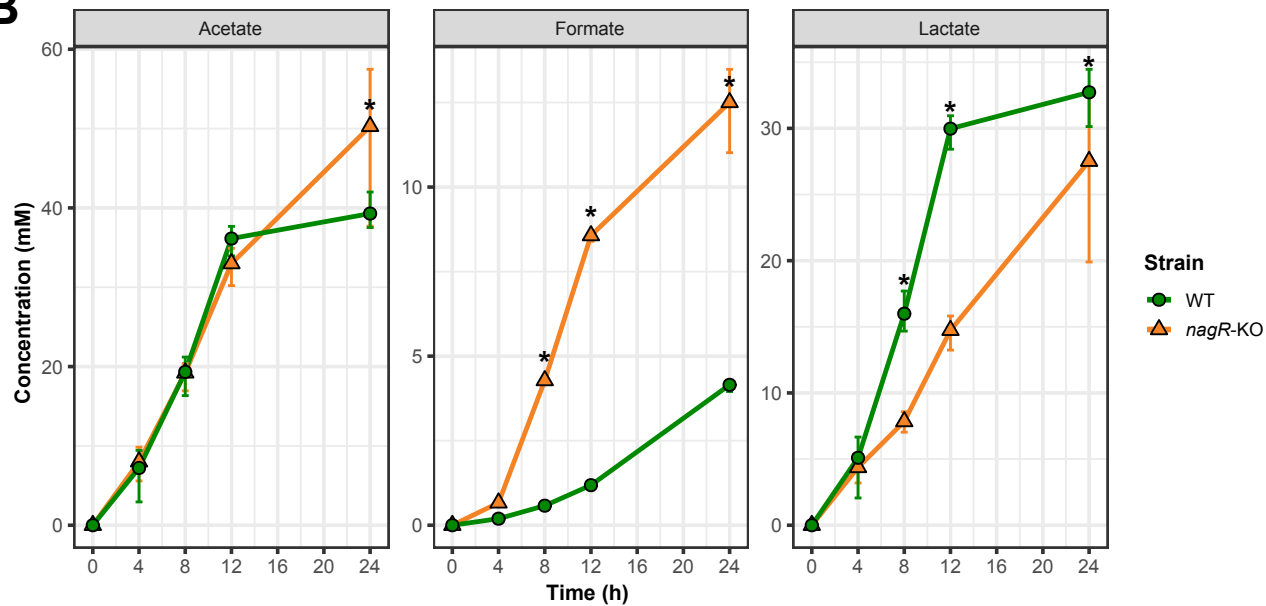

Supplement: FIG S3 [file msystems.00343-22-s0005.pdf]

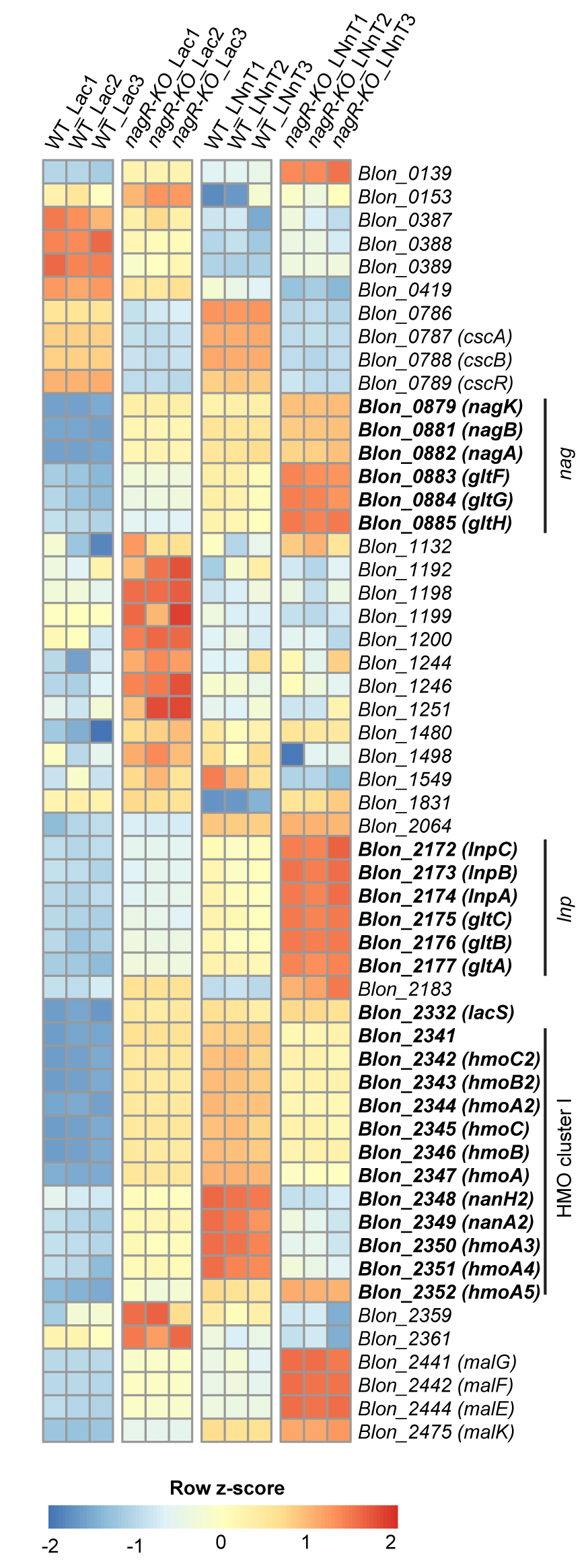

Supplement: FIG S4 [file msystems.00343-22-s0006.tif]

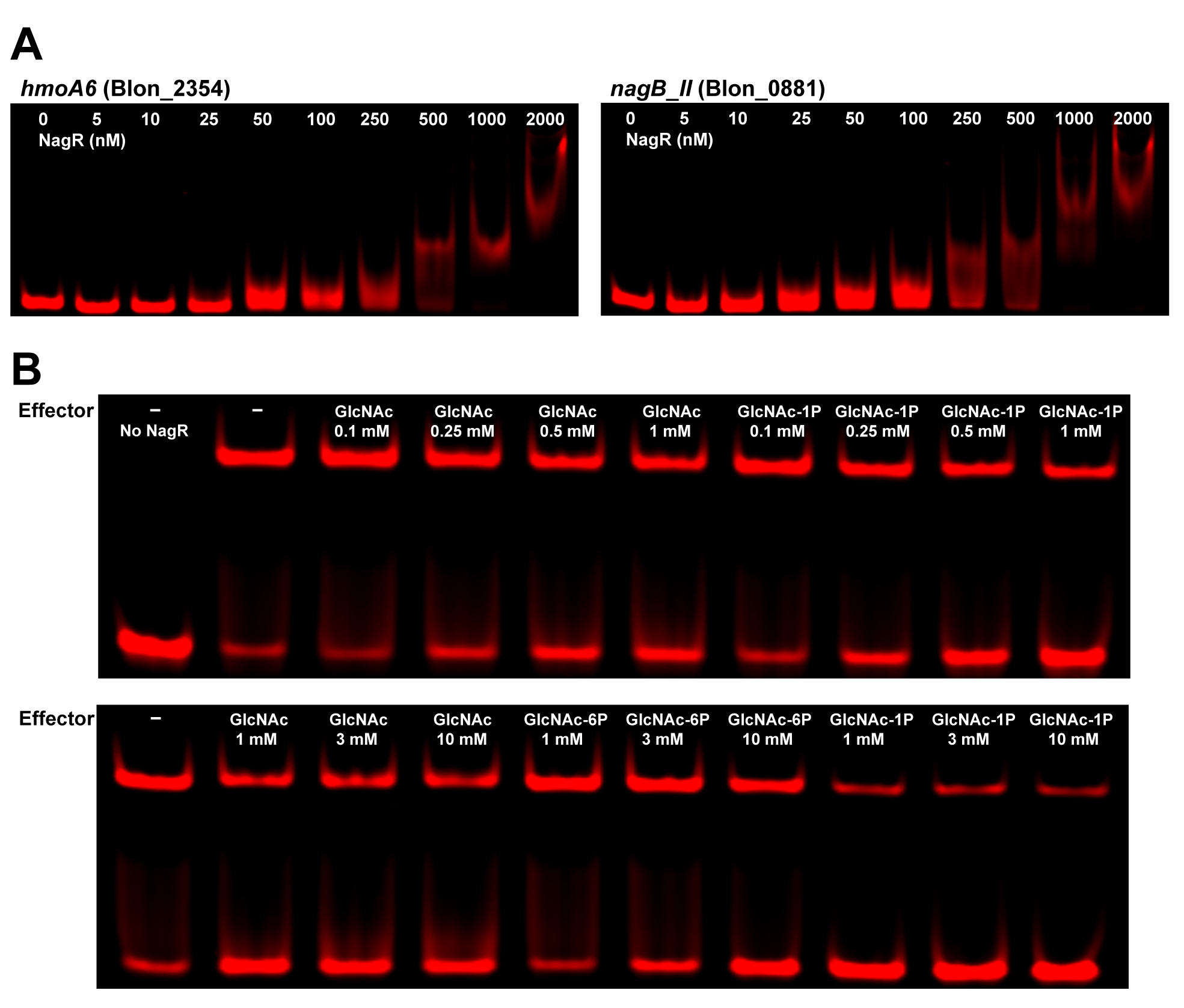

Supplement: FIG S5 [file msystems.00343-22-s0007.tif]
